# Weight and See: Loading Working Memory Improves Incidental Identification of Irrelevant Faces

**DOI:** 10.3389/fpsyg.2012.00286

**Published:** 2012-08-15

**Authors:** David Carmel, Jake Fairnie, Nilli Lavie

**Affiliations:** ^1^Department of Psychology, The University of EdinburghEdinburgh, UK; ^2^Institute of Cognitive Neuroscience, University College LondonLondon, UK

**Keywords:** incidental identification, attention, working memory, load theory

## Abstract

Are task-irrelevant stimuli processed to a level enabling individual identification? This question is central both for perceptual processing models and for applied settings (e.g., eye-witness testimony). Lavie’s load theory proposes that working memory actively maintains attentional prioritization of relevant over irrelevant information. Loading working memory thus impairs attentional prioritization, leading to increased processing of task-irrelevant stimuli. Previous research has shown that increased working memory load leads to greater interference effects from response-competing distractors. Here we test the novel prediction that increased processing of irrelevant stimuli under high working memory load should lead to a greater likelihood of incidental identification of entirely irrelevant stimuli. To test this, we asked participants to perform a word-categorization task while ignoring task-irrelevant images. The categorization task was performed during the retention interval of a working memory task with either low or high load (defined by memory set size). Following the final experimental trial, a surprise question assessed incidental identification of the irrelevant image. Loading working memory was found to improve identification of task-irrelevant faces, but not of building stimuli (shown in a separate experiment to be less distracting). These findings suggest that working memory plays a critical role in determining whether distracting stimuli will be subsequently identified.

## Introduction

As we navigate our complex social and physical environment, we attend to those stimuli deemed most relevant to our current goals. Although we may be aware of goal-irrelevant stimuli to a certain extent, this does not guarantee that we will process them to a level enabling individual identification (Merikle et al., 2001). What determines whether such identification will occur – for example, whether we will be able to later identify a suspect in a crime we had not been expecting to witness? Moreover, to what extent would such incidental identification depend on whether our mind was occupied with other information?

Load theory (Lavie, 1995, 2005) makes specific predictions concerning the interplay of attention, perception, and working memory in determining the processing of task-irrelevant stimuli. When people attend to a task in which certain stimuli are relevant and others are not, a high level of perceptual load will reduce processing of the task-irrelevant stimuli because perceptual capacity is exhausted. In contrast, loading working memory during such a task reduces the ability to actively maintain current stimulus processing priorities (preferentially processing relevant stimuli and suppressing irrelevant ones), thus leading to increased processing of irrelevant stimuli.

In line with the predictions of load theory, numerous studies have demonstrated that high perceptual load reduces behavioral interference caused by task-irrelevant distractors (e.g., Lavie, 1995; Lavie and Cox, 1997) as well as the neural activity associated with distractors (e.g., Rees et al., 1997; Schwartz et al., 2005), whereas high working memory load increases distractor processing in both measures (De Fockert et al., 2001; Lavie et al., 2004; Lavie and De Fockert, 2005). These studies, however, employed indirect measures that do not assess explicit distractor identification.

In studies that employed more direct measures, increasing perceptual load has been shown to impair explicit detection of task-unrelated stimuli (Cartwright-Finch and Lavie, 2006; Carmel et al., 2007, 2011; Macdonald and Lavie, 2008), and reduce explicit identification of meaningful, task-irrelevant objects and faces (Jenkins et al., 2005; Lavie et al., 2009). Conversely, increasing working memory load has recently (De Fockert and Bremner, 2011) been shown to improve detection of a meaningless task-irrelevant stimulus (a square) in a study of inattentional blindness (cf. Fougnie and Marois, 2007). However, the effects of working memory load on incidental identification of meaningful, task-irrelevant stimuli (such as those encountered in real life – for example, in cases that require eye-witness testimony) are not known.

We hypothesized that loading working memory would improve incidental identification of irrelevant stimuli. Although the suggestion that a harder task should result in such improved identification may appear counterintuitive, it follows directly from load theory: if loading working memory impairs processing priorities, then under conditions in which both target and distractor are perceived and compete for further processing (such as that enabling individual-level identification), high working memory load should impair prioritization, thus increasing task-irrelevant processing and improving incidental distractor identification.

To test this prediction, we asked participants to perform a word-categorization task (categorizing names as singers or politicians in Experiment 1, and nouns as kitchen or garden tools in Experiments 2 and 3) while ignoring task-irrelevant distractor images (faces in Experiments 1 and 2, buildings in Experiment 3). This was done during the retention interval of a working memory task, under either low or high load (defined by memory set size: one digit under low load, six under high load). Following the final, critical trial, a surprise question assessed whether the participant could identify the distractor.

## Experiment 1

### Materials and methods

#### Participants

Sixty-two volunteers were recruited at the London Science Museum’s Live Science exhibit. Only participants who were able to recognize the critical trial’s distractor in a control trial were included in the analyses. As the experiment involved two separate tasks (working memory and name categorization), it was essential to verify that participants were engaged in both tasks on the final, critical trial. Twelve participants who responded incorrectly to one of the tasks on the critical trial were thus excluded from the analysis. The remaining 50 participants (30 females, *M* age = 33.7) were randomly assigned to either the low or high working memory load condition (25 per working memory load group). Participants in all the experiments had normal or corrected-to-normal vision.

#### Stimuli and procedure

The experiment was conducted in a quiet, dimly lit area of the museum. Stimuli were created and presented using Matlab 6.5 (MathWorks, Inc.) running Cogent 2000 (Wellcome Department of Imaging Neuroscience, London, UK) on a PC connected to a Sony 15″ monitor. A viewing distance 57 cm was maintained using a chin-rest. All letters and numbers were presented in black Arial font on a white background.

**Figure 1:**
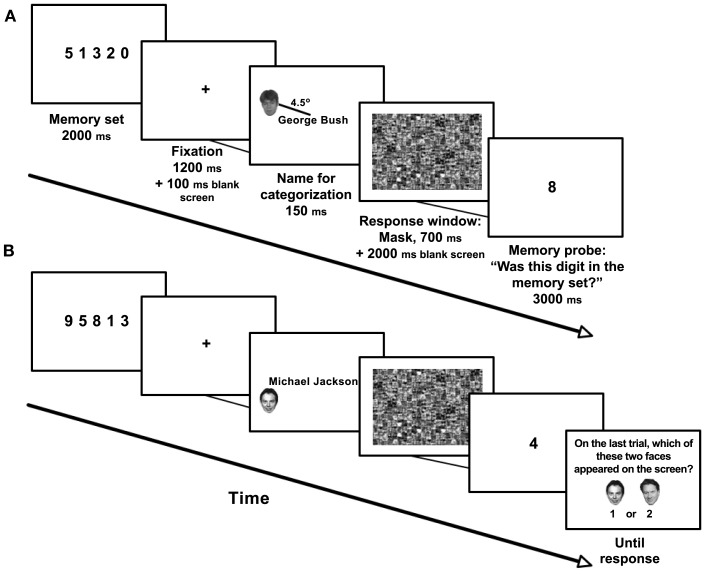
**Experiment 1: stimuli and procedure for (A) non-critical and (B) critical trials**. While maintaining a set of digits in working memory, participants categorized a name as a singer or politician. A task-irrelevant, anonymous distractor face was also presented. Following this, participants were shown a single digit and asked whether it had been included in the memory set. On the critical trial, the face was famous. Following this trial, participants were unexpectedly asked to identify the face they had just seen. Examples shown are of high working memory load trials. Under low working memory load, the memory set consisted of a single digit.

Figure 1A shows the trial sequence. A “Get Ready” prompt was presented at fixation for 1500 ms and replaced by the memory set, which was displayed for 2000 ms. In the high working memory load condition, the memory set consisted of five different digits (digit size 1° × 0.7°, digit array width 4.5°) chosen randomly from the range 0–9, and constrained so that no three adjacent digits could be consecutive (either in ascending or descending order). Under low working memory load the memory set consisted of a single digit at fixation.

The memory set was replaced by a fixation cross (1200 ms), followed by a blank screen (100 ms), and then a 150 ms display containing a famous name at fixation (letter size 0.6° × 0.4°) and an anonymous face in the periphery. The name was either that of a singer (e.g., Mick Jagger) or politician (e.g., George Bush). Participants were instructed to indicate the name’s category and were informed that the face was irrelevant. Anonymous faces were collected from various online resources, cropped, resized (1.5° × 1.5°) and presented in grayscale in one of four equally probable locations (4.5° diagonally from fixation). The name display was followed by a mask (random mesh pattern; 700 ms) and 2000 ms blank screen; participants gave their response to the name categorization task during this period, using the middle and index fingers of their right hand to press one of two keys (the down or left arrow) on a standard keyboard.

Next, a single digit – the working memory probe – was presented for 3000 ms. Participants reported whether this number was included in the memory set presented at the beginning of the trial, using the middle and index fingers of their left hand to press one of two keys (“z” or “x”). Probes were equally likely to have been present or absent in the memory set. The full duration of the response windows for both tasks elapsed regardless of when or whether a response was made.

Participants performed 16 practice trials, followed by 23 experimental trials with anonymous faces. On the 24th, critical trial (Figure 1B), the distractor was a famous face (Tony Blair, British prime-minister at the time, whose name was not used in the name categorization task), and the name was that of a singer. Participants were then given a surprise two-alternative-forced-choice (2AFC) question asking which of two stimuli had just been presented (a 2AFC was used to prevent adoption of a strict response criterion, whereby participants would refrain from offering any response; the other face was Michael Portillo, a well-known politician and TV personality). Finally, in a control trial that was identical to the critical trial, participants were instructed to ignore the memory and categorization tasks and simply observe the screen. At the end of this trial they were again asked the same 2AFC question as in the critical trial, in order to confirm that the critical stimulus was indeed identifiable and verify that any failure to identify the critical stimulus on the critical trial could be attributed to inattention rather than to an inability to identify the distractor. In debriefing participants were asked whether they had ever taken part in a similar experiment, and whether they had been expecting to be asked about the face at some point (all participants answered negatively).

### Results and discussion

#### Working memory task

Mean reaction times (RTs) were significantly slower under high (*M* = 1194 ms) compared to low working memory load [*M* = 980 ms; *t*_(48)_ = 3.945, *p* < 0.001]. Participants were also less accurate under high (*M* = 92.3%) compared to low working memory load [*M* = 96.1%; *t*_(48)_ = 2.017, *p* = 0.024], confirming that the working memory load manipulation was effective and ruling out a speed-accuracy tradeoff.

#### Categorization task

No significant difference was found between the working memory load conditions for either mean RTs [high load *M* = 964 ms, low load *M* = 981 ms; *t*_(48)_ = 0.26, ns] or accuracy [high load *M* = 93.7%, low load *M* = 91.3%; *t*_(48)_ = 1.25, ns], indicating that participants were not differentially engaged in the categorization task in the different working memory load conditions.

#### Incidental identification

**Figure 2:**
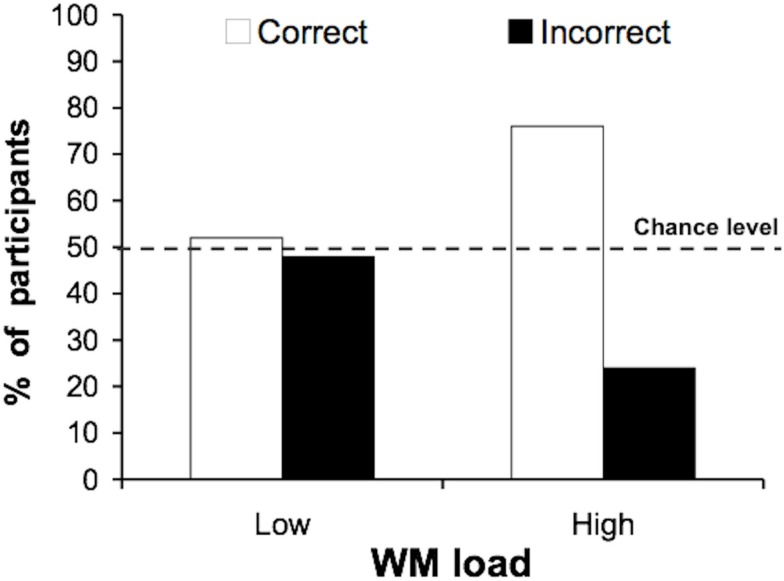
**Experiment 1: incidental identification of the famous face**. Participants performed a name categorization task (singer/politician) while ignoring peripheral faces. Significantly more participants identified the famous face correctly under high working memory load than under low working memory load.

As predicted, loading working memory improved identification of the irrelevant face. Significantly more participants correctly identified the face under high (76%), compared to low (52%) working memory load [Figure 2; $\chi_{\left(1\right)}^{2}$ = 3.125; *p* = 0.039]. These results are consistent with our prediction that under low working memory load, efficient control of stimulus processing priorities would prevent processing of the peripheral face’s identity, whereas under high working memory load prioritization would be impaired, increasing incidental identification of the famous face.

## Experiment 2

In Experiment 1, stimulus competition on the critical trial was maximized by presenting a politician’s face with a singer’s name, potentially leading to response competition. Could the effect of working memory load on identification be restricted to cases in which response competition occurs? To test this possibility, in Experiment 2 we presented the same distractor faces used in Experiment 1, but now asked participants to categorize words as either garden (e.g., wheel barrow) or kitchen (e.g., frying pan) tools. If the effect of working memory load is due to impaired processing prioritization under high load, it should be found again here, even for irrelevant stimuli that do not directly compete with the required response. If, however, the effect of working memory load is due to response competition then it should not be found.

### Materials and methods

#### Participants

In this and all subsequent experiments participants were recruited from the UCL subject pool, and paid £5 for participation. Of 47 volunteers in this experiment, 9 were excluded from analysis for responding incorrectly to one of the tasks on the critical trial. The remaining 38 participants (19 per working memory load group; 20 females) had a mean age of 24.7.

#### Stimuli and procedure

These were identical to Experiment 1, except for the following differences. The experiment was conducted in a small, dimly lit testing room in UCL’s Psychology Department. On the categorization task, singer and politician names were replaced with garden and kitchen tool names.

### Results and discussion

#### Working memory task

Mean RTs were again significantly slower under high (*M* = 1180 ms) compared to low working memory load [*M* = 994 ms; *t*_(36)_ = 1.867, *p* = 0.035]. Participants were also less accurate under high (*M* = 94.1%) compared to low working memory load [*M* = 97.1%; *t*_(36)_ = 1.842, *p* = 0.037].

#### Categorization task

No significant difference was found between the working memory load conditions for either mean RTs [high load *M* = 1005 ms, low load *M* = 1070 ms; *t*_(36)_ = 0.619, ns] or accuracy [high load *M* = 96.4%, low load *M* = 94.9%; *t*_(36)_ = 0.809, ns].

#### Incidental identification

**Figure 3:**
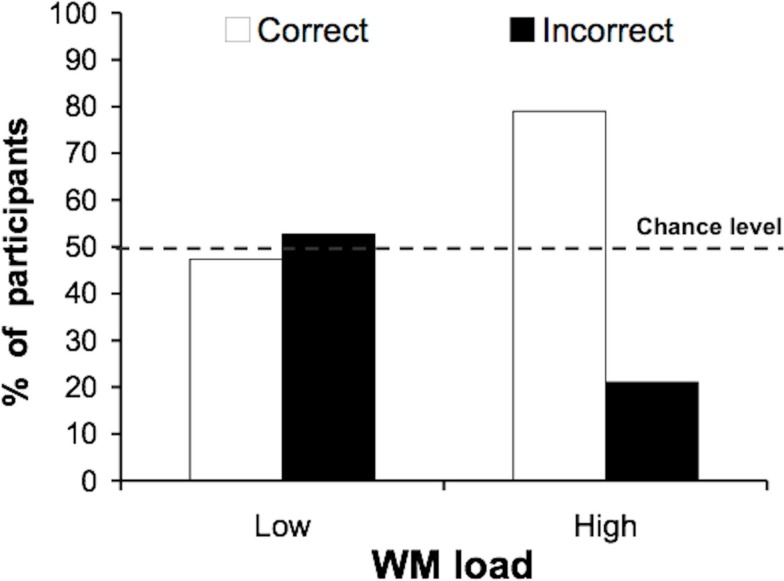
**Experiment 2: incidental identification of the famous face**. Participants performed an object categorization task (garden/kitchen object) while ignoring peripheral faces. Again, significantly more participants identified the famous face correctly under high working memory load than under low working memory load.

As in Experiment 1, loading working memory improved identification. Significantly more participants correctly identified the face under high (79%), compared to low (47%) working memory load [Figure 3; $\chi_{\left(1\right)}^{2}$ = 4.07; *p* = 0.022]. This result demonstrates unequivocally that the effect found in Experiment 1 was not due to increased response competition; rather, finding this effect again using task categories unrelated to the distractor suggests that loading working memory causes a general impairment to stimulus processing priorities, thus leading to increased rates of distractor identification.

## Experiment 3

Active maintenance of stimulus-priorities in working memory is needed when task-irrelevant stimuli would naturally be given high priority, but the task requires that they should be ignored. Faces are known to be a high-priority class of stimuli, and can be particularly hard to ignore (e.g., Lavie et al., 2003). Conversely, irrelevant stimuli that are not inherently given high priority and are thus less likely to attract attention should impose little, if any, demands on the working memory system that maintains current processing priorities when distracting stimuli are present. The effects of working memory load should therefore be restricted to distractor stimuli that compete with task-relevant stimuli despite their irrelevance to the present task. Indeed, Yi et al. (2004) failed to find an effect of working memory load on neural responses to irrelevant images of places, whereas De Fockert et al. (2001) did find such an effect for faces. In Experiment 3 we therefore examined the effect of working memory load on incidental identification of buildings – a class of stimuli visually comparable to faces (e.g., both involve configural processing and can be identified at the level of individual tokens) but not known to be inherently given high priority.

### Materials and methods

#### Participants

Thirty-one new participants (17 female; *M* age = 21.9) were recruited and randomly allocated to either the high (16 participants) or low (15 participants) working memory load condition.

#### Apparatus, stimuli, and procedure

These were the same as in Experiment 2, except for the following differences. Instead of faces, the peripheral stimuli were pictures of buildings. On all trials except the critical and control trials, these were non-famous buildings collected from various online resources, cropped to 1.5–2° (width) × 1.5° (height) and presented in gray scale. On the critical and control trials, the peripheral image was of a famous building – either the White House or Buckingham Palace (counterbalanced across participants and working memory conditions).

### Results and discussion

#### Working memory task

The working memory manipulation was again effective: mean RTs were significantly longer under high (*M* = 1072 ms) compared to low working memory load [833 ms; *t*_(29)_ = 2.512, *p* = 0.009]. Accuracy rates did not differ significantly between the low (*M* = 93.1%) and high working memory load [94.3%; *t*_(29)_ = 0.606, ns].

#### Categorization task

There were no significant differences between the working memory load conditions for either mean RTs [high load *M* = 1038 ms, low load *M* = 1006 ms; *t*_(29)_ = 0.303, ns] or accuracy [high load *M* = 94.3%, low load *M* = 92.6%; *t*_(29)_ = 0.484, ns].

#### Incidental identification

**Figure 4:**
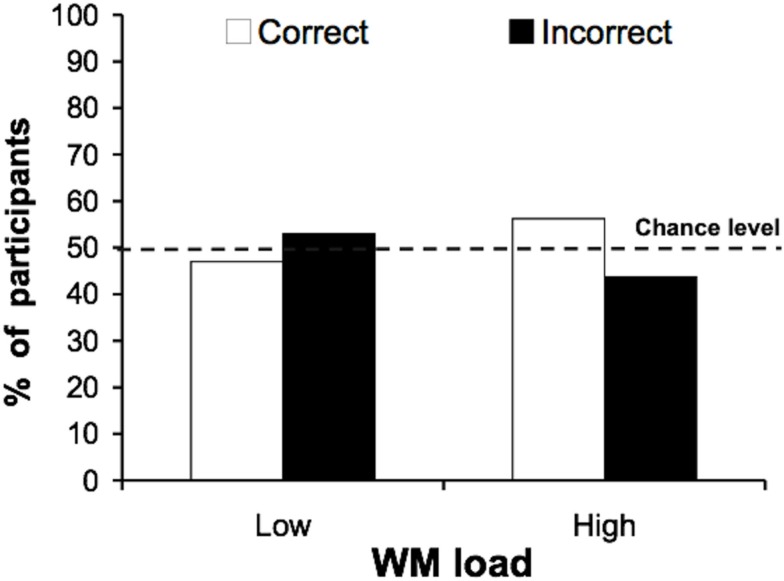
**Experiment 3: incidental identification of the famous building**. Participants performed an object categorization task (garden/kitchen object) while ignoring peripheral buildings. This time incidental identification of a famous building was at chance under both low and high working memory load.

Unlike previous experiments, performance on the critical trial did not differ between high (56%) and low (47%) working memory load [Figure 4; $\chi_{\left(1\right)}^{2}$ = 0.285, ns]. This finding supports our hypothesis that loading working memory improves identification of highly distracting faces, but not of less distracting buildings. The contrast between the results of Experiments 1 and 2 and those of Experiment 3 indicates that the effect of working memory load depends on the type of distractors presented, ruling out general effects of working memory load (e.g., task difficulty or spatial extent of attention in different working memory load conditions).

It is worth noting that no participants were excluded from the analysis for responding incorrectly on the critical trial to either the working memory or categorization tasks in this experiment, whereas nine participants were excluded in Experiment 2, which had an identical categorization task. This difference indirectly supports the suggestion that faces are more distracting than buildings: in Experiment 2, the presence of a famous face in the periphery may have caused a level of distraction that impaired task performance, whereas the presence of a famous building in Experiment 3 did not. An alternative interpretation of this difference is that the famous buildings used in Experiment 3 were simply less identifiable than the famous face used in Experiments 1 and 2, but this possibility is ruled out by the fact that all participants were able to identify the buildings in the control trial.

## Experiment 4

Using the same working memory and word-categorization tasks across experiments, we found that loading working memory enhances identification of distractor faces (Experiment 2) but not distractor buildings (Experiment 3). We hypothesized that this difference is mediated by these categories’ different distraction potency, which requires differential recruitment of cognitive control by working memory. Indeed, previous research (Jenkins et al., 2003; Lavie et al., 2003) has suggested that faces are more distracting than other categories. To establish that faces are indeed more distracting than buildings in the present experimental context, in Experiment 4 we employed the same categorization task used in Experiments 2 and 3, and compared the RT distractor interference effects caused by faces and buildings.

### Materials and methods

#### Participants

Twenty-six new volunteers (13 female, *M* age = 24.9) took part in this experiment.

#### Stimuli and procedure

Only the object categorization task was used in this experiment. On each trial, the object name and distractor were presented for 150 ms, followed by a 700 ms mask and 2000 ms blank screen, which together comprised the response window. The distractor could be a famous face (Tony Blair, 10% of trials); a famous building (the White House, 10% of trials); an anonymous face (40% of trials); or a non-famous building (40% of trials; we selected 32 anonymous faces and 32 non-famous buildings from the sets used in the previous experiments). All pairings of target category, distractor type and distractor location were counterbalanced in randomized order within each 80 trial block. Participants performed a 16 trial practice followed by three experimental blocks. At the end of the experiment, participants were shown the pictures of Tony Blair and the White House and asked to name them. All did so correctly.

### Results and discussion

**Figure 5:**
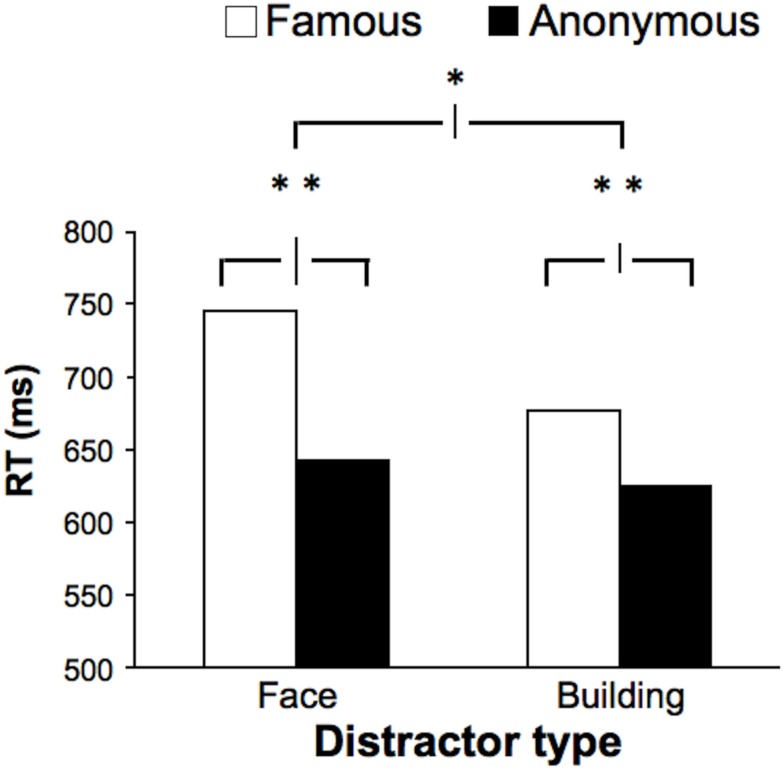
**Experiment 4: distractor interference from famous faces and buildings**. Participants performed an object categorization task (garden/kitchen object) while ignoring peripheral images of faces and buildings. Though they were ignored, these distractors affected RTs – famous faces and buildings both slowed responses compared to anonymous exemplars, but the magnitude of the effect for faces was double that for buildings, showing that faces are more distracting. Error bars = 1 standard error of the mean within-subject difference between famous and anonymous (lower two bars) and between the famous-anonymous differences of the different categories (upper bar). **p < 0.01; *p < 0.05.

A 2 (fame: famous, anonymous) × 2 (category: faces, buildings) repeated-measures ANOVA of RTs revealed main effects of both fame [*F*_(1, 25)_ = 10.102, *p* = 0.004], and category [*F*_(1, 25)_ = 12.293, *p* = 0.002]. RTs were longer in the presence of famous (vs. anonymous) items and in the presence of faces (vs. buildings; Figure 5). Importantly, a significant interaction [*F*_(1, 25)_ = 4.942, *p* = 0.035] confirmed that although both of the famous distractor types produced significant interference effects compared to anonymous images [*M* = 744 vs. 642 ms for the famous and anonymous faces, respectively, *t*_(25)_ = 2.986, *p* = 0.006; *M* = 675 vs. 625 ms for the famous and anonymous buildings, respectively, *t*_(25)_ = 3.161, *p* = 0.004] the distractor interference effect was greater for faces (mean distractor effect: 102 ms) than for buildings (mean distractor effect: 50 ms). Accuracy rates were high and did not vary between conditions (*M* accuracy = 91–92% in all conditions).

## General Discussion

The experiments presented here demonstrate that loading working memory can improve incidental identification of task-irrelevant stimuli. This improvement, however, depends on how distracting the irrelevant stimuli are: Loading working memory improved identification of irrelevant faces (Experiments 1 and 2), but not irrelevant buildings (Experiment 3), which were shown to be less distracting (Experiment 4). Indeed, the finding that buildings are less distracting than faces also suggests an explanation for the contrast between the results of previous studies, which found that loading working memory enhances neural responses to face distractors (De Fockert et al., 2001) but not to background images of places (Yi et al., 2004). Incidental identification of an irrelevant stimulus thus appears to depend on the interplay between distraction potency and top-down attentional processes.

The present results attest to a fundamental operating principle of attention: competition between stimuli for processing is actively managed by executive control functions involving working memory. High working memory load reduces control over processing priorities, leading to a greater likelihood of processing task-irrelevant information. Whereas previous studies (De Fockert et al., 2001; Lavie et al., 2004) have demonstrated that working memory load affects the extent to which response-competing distractors interfere with task performance, indirectly indicating increased processing of distractors under high working memory load, here we show that increasing working memory load affects an explicit, direct measure of processing, improving incidental identification of irrelevant information.

### Types of memory load and their effects on visual processing

Another recent study (De Fockert and Bremner, 2011) also used a direct measure to address a related question. Rather than investigating whether loading working memory would affect identification of an irrelevant stimulus whose presence was expected, as we did here, De Fockert and Bremner (2011) examined the effect of working memory load on inattentional blindness – a failure to notice the presence of an unexpected stimulus during performance of a perceptual task. On each trial, they had participants judge which of the two arms of a cross was longer; on the final experimental trial they presented an additional, unexpected critical stimulus (a small square). They then asked participants whether they had seen the critical stimulus, and – in line with the predictions of load theory – found reduced inattentional blindness (i.e., improved stimulus detection) under high working memory load.

Why did loading working memory enhance the detection of a relatively meaningless critical stimulus (a square) in De Fockert and Bremner’s (2011) study, whereas here we found that working memory load only affected identification of more distracting faces but not less distracting buildings? This seeming discrepancy can be accounted for by pointing out that our irrelevant stimuli were expected, whereas De Fockert and Bremner’s (2011) were not. Unexpected stimuli are more likely than expected stimuli to compete with the target for attention, even when they are meaningless (e.g., Forster and Lavie, 2008). For expected stimuli to capture attention, however, they must be particularly distracting or meaningful. Famous face distractors therefore remain attention capturing and compete with the target, requiring cognitive control mediated by working memory, even when they are expected (as demonstrated by Experiment 4, as well as Jenkins et al., 2002; Lavie et al., 2003). In contrast, expected buildings were not distracting enough to compete with relevant stimuli. Consistent with this interpretation, the effect of working memory load on detection of meaningless stimuli disappeared in De Fockert and Bremner’s study when participants were warned that an additional stimulus would appear (De Fockert and Bremner, 2011, Experiment 2) and Macdonald and Lavie (2008) found no effect of working memory load on detection rates of an expected meaningless stimulus (an abstract “squiggle”).

According to load theory, in order for working memory load to have the predicted effect it is necessary for stimulus competition to occur, so that active prioritization by executive control is required. Indeed, in the present study the irrelevant stimuli were presented simultaneously with the categorization task stimuli and competed with them; and the unexpected stimulus in De Fockert and Bremner’s (2011) study competed with the attention task stimulus, requiring prioritization by working memory. In contrast, a different study (Fougnie and Marois, 2007) examined detection rates for an unexpected stimulus (a small clover shape) that was presented on its own (with no additional task and no competition from other stimuli) during a memory task’s retention interval. As there was no stimulus competition and no need to prioritize processing of one stimulus over another, loading working memory would not be expected to enhance detection of the unexpected stimulus in this case.

Indeed, Fougnie and Marois (2007) found no improvement in detection rates for the unexpected stimulus under higher memory load. In fact, they found that increasing memory load led to lower detection rates. Why would increasing memory load impair detection? This can be explained by considering the specific memory task used. Participants in Fougnie and Marois’s (2007) study were asked to either remember a set of letters in the order they were given (low load), or rearrange the letters in alphabetical order in their minds (high load). In both cases, following a delay period, participants had to decide whether a probe letter that was presented in a particular spatial location (above one of several lines drawn along the horizontal midline) was in the correct place in the memorized sequence. Basing the test on the memory probe’s spatial location, as well as the requirement to transform the memory set into a specific alphabetic left-to-right sequence, are highly likely to involve visual short-term memory (VSTM) in the high load condition. Unlike executive control working memory processes, VSTM draws on the same resources that are required for sensory processing (Pasternak and Greenlee, 2005), and activates similar visual cortex representations to those involved in stimulus encoding (Harrison and Tong, 2009; Serences et al., 2009). Consistent with this, recent findings (Konstantinou et al., 2012) have demonstrated a dissociation between the effects of loading VSTM and those of loading executive control working memory processes. Loading VSTM was found to have similar effects to those of perceptual load, reducing perception of low priority stimuli. Furthermore, Konstantinou et al. (2012) also showed that as loading VSTM reduces the available capacity for sensory representation, it affects perception regardless of whether there is stimulus competition.

The dissociation between the effects of loading VSTM and those of loading executive control working memory processes also addresses the results of Rose et al. (2005), who used a visual memory n-back task in which participants had to detect on-screen repetitions of target stimuli that were separated by either no (1-back, low load) or one (2-back, high load) stimulus, while ignoring irrelevant background pictures. The findings of Rose et al. (2005) indicated reduced processing of the background images under high load. The n-back task’s requirement to detect on-screen repetitions, however, places demands on VSTM: it engages perceptual encoding and comparison processes simultaneously and continuously, as participants have to perceive and encode new stimuli rapidly and compare them to templates stored in memory. Increasing the load of such a task thus placed greater demands on VSTM, which in turn led to reduced processing of the ignored background images. In contrast, the type of working memory task used in the present study isolates the effect of maintaining stored information by presenting the irrelevant stimuli during a retention interval in which the memory set is neither updated, nor compared to incoming visual stimuli.

In a more recent study, Klemen et al. (2010) used a similar experimental design to that of Rose et al. (2005), but employed an auditory n-back task and again found reduced processing of ignored images under high load. Although the memory manipulated in this study was not visual, the task again required participants to continuously engage in perceptual encoding of new information. Auditory perceptual encoding can interfere with visual encoding and vice versa (Jolicoeur, 1999), and several recent studies (Sinnett et al., 2006; Santangelo et al., 2007; Brand-D’Abrescia and Lavie, 2008; Macdonald and Lavie, 2011) have demonstrated crossmodal interference effects between vision and audition. The results of Klemen et al. (2010) may thus also be due to increased load on perceptual encoding (similar to the effects of perceptual load) rather than on the executive control processes manipulated in the present study.

Of course, it must be acknowledged that every task involves a component of executive control, which is required for participants to remain focused on the task at hand. However, the above discussion implies that the relative extent to which different manipulations place demands on executive control working memory processes versus VSTM may determine the perceptual outcome of each manipulation. Although it is plausible that the type of working memory manipulation employed in the present and previous studies (De Fockert et al., 2001; Lavie et al., 2004; Lavie and De Fockert, 2005; De Fockert and Bremner, 2011; Weil et al., 2011) would tax executive control (with very little effect on VSTM) whereas the manipulations used by Fougnie and Marois (2007), Rose et al. (2005), and Klemen et al. (2010) would place higher demands on VSTM (with a relatively smaller effect on executive control), the experiments presented here were not designed to address this issue. Konstantinou et al. (2012) have taken an important first step toward clarifying the differential effects of executive control working memory and VSTM manipulations, but the issue remains an important avenue for further research.

### The selectivity of the working memory load effect

In the present experiments, loading working memory altered incidental identification of task-irrelevant faces without affecting performance on the attentional word-categorization task. This selective effect of working memory load has been reported in several previous studies using similar tasks (De Fockert et al., 2001; Lavie et al., 2004; Lavie and De Fockert, 2005; Weil et al., 2011), and importantly, indicates that participants did not deploy different levels of attention to the categorization task under different working memory load conditions (cf. Kin et al., 2005).

If perception has limited capacity, why didn’t the improved face identification come at the expense of performance on the categorization task? This question can be addressed by considering the nature of the categorization task, which involved classifying a single, highly familiar person or object name presented at fixation, and thus imposed low perceptual load. Therefore, even when the irrelevant face was identified, performance of the categorization task is unlikely to have exhausted perceptual resources and so was unimpaired.

The improved incidental identification of faces under high working memory load is therefore not due to a change in perceptual capacity, but to the reduced availability of executive control: Load theory posits that executive control is an active mechanism (Lavie et al., 2004; Lavie, 2005) that enables one to distinguish relevant from irrelevant stimuli (under conditions of low perceptual load, in which both kinds are perceived) and to effectively ignore the irrelevant stimuli. Such active ignoring (suppression of irrelevant stimulus representations) is adaptive as it prevents further processing that could lead to response conflict, and it thus prevents the individual-level identification investigated here. Under high working memory load this active ignoring is impaired, making the face available for identification.

### Implications for daily life

The role established here for working memory in the identification of meaningful stimuli has the ironic implication that a highly adaptive ability (allocating processing priorities in the face of competing stimuli) may lead us to miss meaningful information when it operates at full capacity. In daily life, reduced executive control over task performance is seen as undesirable. Here we show, however, that it could also have advantageous consequences when irrelevant information turns out to be pertinent after all (see also Olivers and Nieuwenhuis, 2005, for a related idea regarding the attentional blink). A classic example of information surprisingly becoming relevant comes from the field of eye-witness testimony. A witness’s ability to identify a person who had previously been irrelevant to their current goals is vital for the accuracy of their testimony. Interestingly, our findings suggest that a state of high working memory load should not undermine, but if anything improve, the validity of the witness’s statement. In today’s busy world one often encounters a wealth of meaningful information while their working memory is heavily engaged (for example while using a mobile phone, which can provide a steady flow of information, or simply while thinking and planning). Elucidating the role of working memory load in the processing of meaningful information, and in the ability to recognize its specific content, therefore not only advances scientific knowledge but also our understanding of information processing in daily life.

## Conflict of Interest Statement

The authors declare that the research was conducted in the absence of any commercial or financial relationships that could be construed as a potential conflict of interest.
